# What prevent women for a sustainable use of maternal care in two medical districts of Burkina Faso? A qualitative study

**DOI:** 10.11604/pamj.2014.18.43.2210

**Published:** 2014-05-12

**Authors:** Donmozoun Télesphore Somé, Issiaka Sombie, Nicolas Meda

**Affiliations:** 1 Société d'Etudes et de Recherche en Santé Publique, Ouagadougou (SERSAP), Burkina Faso; 2 West African Health Organization (WAHO), Bobo-Dioulasso, Burkina Faso; 3 Centre MURAZ, Bobo-Dioulasso, Burkina Faso

**Keywords:** Maternal care, skilled care, rural, medical district, Burkina Faso

## Abstract

**Introduction:**

Skilled attendance is one of the major strategies to curtail maternal mortality, specifically in developing countries. Despite the low level of equipment, it is only in health facilities that skilled care are provided during pregnancy and childbirth; but there are some barriers which prevent women to use health facilities for good care.

**Methods:**

This study was carried out in Ouargaye where a skilled care initiative was implemented by Family care International with the aim to increase the skilled attendance at delivery and Diapaga, the control district. Thirty (30) In-depth interviews, 8 Focus group discussions and 6 non participant observations were carried out. Participants were women from 15-49 years. All the interviews were tape-recorded, transcribed and analysed line by line. NVIVO was used to manage the interviews.

**Results:**

Four types of barriers have been described by women; 1) the cultural barriers concern the low status of women in the two districts and some traditional beliefs which mean that women can not always decide to use health facility by themselves. 2) The geographical barrier is about the distance to reach health facility and the lack of transport means. 3) The financial barrier to pay care and drugs. 4) Bad organization of care and poor quality of care provided to women.

**Conclusion:**

To minimize the risk of complications during pregnancy and childbirth, it is important that women use health facilities. The barriers described by women are not insurmountable but needed to be integrated in a global comprehensive health policy.

## Introduction

WHO estimated maternal mortality ratio of 920/100000 live births for Sub-Saharan Africa with a lifetime risk of a maternal death of 1 in 16 [1]. These rates are very high when compared to developed countries lifetime risk of 1 in 2800 estimated during the same time period [1].

In Burkina Faso, the maternal mortality ratio is around 484/100 000 live births [2] due to low access to skilled care during pregnancy and childbirth.

The reasons for these deaths are known but there is not yet a cost-effective strategy which takes account of the economical, social and political context of the country. Many strategies have been tried but the mortality rate still remains high. It is known since a long time that skilled attendant at delivery could contribute to a significant reduction of maternal mortality [3]. Some experiences of significant reduction of maternal mortality have shown that skilled attendance at delivery is an important strategy. Skilled attendant is defined as “*an accredited health professional -such as a midwife, doctor or nurse -who has been educated and trained to proficiency in the skills needed to manage normal (uncomplicated) pregnancies, childbirth and the immediate postnatal period, and in the identification, management and referral of complications in women and newborns*” [1].

It is assumed that most of maternal and newborns deaths occurs around the time of childbirth or shortly thereafter. 80% of maternal deaths are due to a few direct obstetric complications: Sepsis, haemorrhage, eclampsia, obstructed labour and unsafe abortion. Most could be prevented or managed if the woman had access to a skilled attendant with the necessary back-up and support [3]. It is also shown that in sub-saharan Africa, 26.8% childbirths are managed by relatives. Only 5, 8% are managed by doctors. Clearly, skilled attendance is important for the reduction of maternal deaths. But, in Burkina, health facilities use rate is around 34, 06% [4]. The consequences of this low use of health facilities are that some women delivered at home without a skilled attendant [5] and increase the risk of a severe complication or of dying. In the context of Burkina, there are some barriers which prevent women from using health facilities for skilled care.

To understand these barriers, some quantitative studies have already described users and non users’ perceptions of the quality of care in current health services in Burkina [6, 7]. But women perceptions of low utilization of health facilities are not well documented. Some studies have pointed the financial barrier to be a major factor but we think this is a limitative reason due to the general context of poverty in developing countries.

This article presents the results of a qualitative study carried out in the Centre eastern (Ouargaye) and Eastern region (Diapaga) of Burkina to reach a better understanding of how people perceive skilled care. The study was a component of a safe motherhood strategy evaluation. The skilled care initiative (SCI) was implemented by Family Care International (FCI) with the aim of increasing the rate of skilled attendance at delivery in Ouargaye [8]. The study was a qualitative study addressing the demand side with the aim of identifying and understanding the barriers to and facilitators of use of skilled care during pregnancy and childbirth in the context of the SCI.

## Methods

The study was carried out in two medical districts of Burkina (Ouargaye and Diapaga). Ouargaye and Diapaga were chosen because the study was a part of an evaluation of a safe motherhood strategy implemented by Family Care International. The intervention was the skilled care initiative (SCI). The evaluation was a quasi experimental evaluation with a qualitative part concerning barriers and facilitators to use of skilled care.

In the medical district of Ouargaye, one health centre was chosen in the SCI area and another in non SCI area. In Diapaga, one health centre was also chosen. These health centres were chosen based on the criteria of SCI area or not, the presence of an auxiliary midwife and the distance to the district hospital.

Theoretical sampling [9–11] was used. The number of participants depended at the end of the study on the saturation of some information categories. Thirty in-depth interviews (IDI), 8 focus group discussions (FDG) and 6 non participant observations were performed. The FGD were carried out with 8 to 12 participants in the local language (Yana or Mooré in Ouargaye and Goulmanchema in Diapaga). All interviews (IDI and FGD) were tape-recorded and notes taken during interviews.

QSR NVIVO 2. 0 was used to manage transcripts during analysis.

## Results

**Categories and themes developed:** the categories and themes developed for this study are shown in Table 1.

**Table 1:** Categories and themes developed

Categories	Themes
Community perception of quality of care	Appreciation of care
	Reception in maternity
	Adequacy of resources
	Provider role and referral
Facilitators to use of skilled care	Perception of wellbeing
	Perception of risk of death
	Prior experience
Barriers to use of maternal services	Finances
	Cultural practices
	Bad treatment in maternity services
	distance

**Participants characteristics:** Table 2 whos the main socio demographic characteristics of the study participants

**Table 2:** Participants main socio demographic characteristics

Variable	N=30	Percentage (3%)
**Age**		
[15-24]	11	37
[25-34]	13	43
[35-44]	03	10
[Unknown]	03	10
**Educational level**		
None	16	53
Literate	5	17
Primary	8	27
Secondary	1	3
**District**		
Ouargaye	20	67
Diapaga	10	33

**Figure 1:**
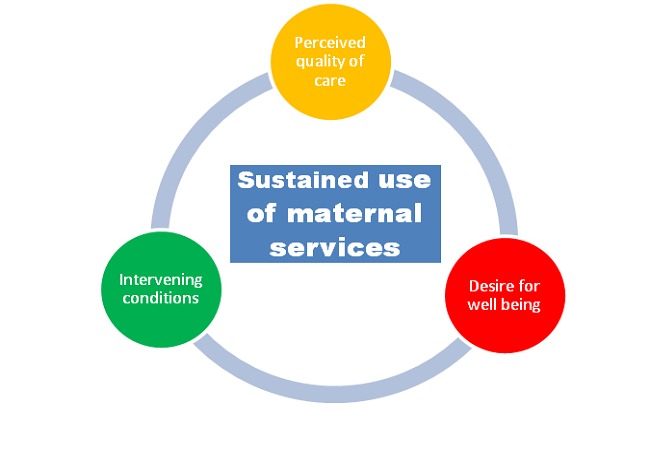
Conceptualization of factors that may affect use of skilled care at delivery

**Study framework**: The conceptualization of factors that may affect use of SCD is shown in Figure 1.

### How quality of care is perceived in both medical districts?

In both medical districts, care in general is appreciated by users in terms of what they gain and how care is given to them in health facilities. The perception of care concerns the service and not really the quality of care received. This could be explained by the low educational level of informants (see informants characteristics). In general, they don't have a long tradition of modern care; so it is difficult some times for them to categorise care as bad or good objectively. The appreciation concerns other aspects, related to the providers. Quality is not a real problem in either district. To appreciate care means that you are satisfied with what you are requesting. If they are well received by providers and they are asked to buy some drugs, it means that the care is good. Providers are perceived as persons who have a secret knowledge and could help them to recover if some thing is wrong. In the particular case of maternity, they do refer to care received at home and care at the maternity. In both districts, they recognized the good quality of maternity care;

*“At home, there is a lot of suffering because, if your child is ill, you can not know; but in maternity, they examine you and your child after delivery to see if you are well; if not, they will make you buy drugs”*. User, village C.

Good or bad perception of care depends on how well users have been received in the maternity for antenatal care or for delivery by health providers. Reception is one of the main elements of appreciation of quality of care for women. Reception is described as negative when the user felt that the provider did not respond to her greetings, shouted at her during weighing or delivery. In both districts, reception is described as good. The resources are also an important element for perceptions of quality of care. The availability or not of adequate supplies, influence the quality of care.

### Barriers to use of maternity services

According to our findings, the use of maternity services is affected by many factors. These factors are related to the health system, the environment and users themselves. (See the conceptual framework for more details).

### Lack of decision-making power

The low status of women concerns many domains (economic, social, political, cultural, etc); in health domain, most of the time, women did not have the power to decide for care seeking for their children and for themselves. A married woman depends entirely on her husband and his family. This is justified by the bride price paid by the man. The man decides all for his wife. The two districts have still kept their traditional values. In Diapaga, women are totally dependant on husbands or husbands’ relatives.

*“If you know that you depend on some one and this person do not give you permission, you can not go out; Oh! If he forbids you, will you do?” User 6, Village C*.

*In both districts, the decision-making process is related to financial capacity. Men have all the power and especially economic power. Harvests from fields go all to the man. So, a woman cannot decide by herself to go to maternity if she has not some money to pay care and drugs*.

*“It is the family chief who does it. If you don't earn anything, it is difficult; if it a case of illness, he will make you better. One says ‘the woman is the home’ but the woman is not the chief of the family. So, it is difficult for her to go by herself to health centre”. User, Village A*.

### Finances

*In the districts, when women have permission to go to health centre, there still some times remains a financial problem. This problem is appreciated differently in the two districts. In Ouargaye district, especially in SCI area, users talk about poverty by making reference to the past. They mentioned also the fact that they now have aid for visit card, some drugs and antenatal care. In the SCI area, there is a waived fee for some care*.

*In non SCI area, there is no aid for care in the maternity and the users have to pay for all the care. For users, the cost is high; so it is not possible for them to pay all the time. Some of them know that there is good quality of care in the health centre, but they cannot go. “All is money; if you go and you have no money, what will you say there? You know that you will not receive free care. You will wait to have money before going to maternity. This is the reason of delay before going to health centre”. User_BR, village B*.

*In Diapaga district, the problem is similarly the same. Users said that they don't have free care in maternity. They have to pay for all care they received*.

### Cultural practices

*“In Ouargaye district, when a woman is pregnant for the first time, she is not able to talk about it or to go to a health centre before a traditional rite. This rite takes place around the third month of the pregnancy. These practices are common to the SCI and Non SCI areas. It is appreciated in the same way. This practice is about the announcement of the pregnancy; it was necessary to announce the pregnancy before the woman goes to the maternity. If this practice was not done, it is not possible to say that this person is pregnant. You just look at and wait the announcement”* User_S_village A.

In Diapaga, some practices prevent women from using maternity services. This practice concerns divination. Many domains of the individual life are built around this belief.

*“I am not “tapeur de sable “(soothsayer); but I see some times women that “tapeur de sable”(soothsayer) refuses that they go out, even to go to maternity for weighing and delivery; she has to deliver at home; (…) People used to go to see “tapeur de sable “(soothsayer); it is not helpful. In these case, the woman will deliver in bad conditions, may be, she can lose her baby or she will died”*. FGD-village C.

This practice is common to the whole community. But, nowadays, some persons specifically Christian and muslim, don't take account of these practices. The persistence of these practices is due to ignorance according to some women.

Other practices preventing women from using skilled care are about the manifestation of courage during childbirth. Women should show their courage by supporting pains. This is not always possible; so, they can decide to stay at home, not to suffer the ridicule of others.

### Bad treatment in maternity service

According to some users, they received bad treatments in maternity services during antenatal care or childbirth. Theses bad treatment some times, discouraged them to go back to maternity. During the study, we observed the interactions between providers and users during antenatal care and the report is that providers do not welcome users nicely in maternity services. They do just what they have to do and some times, the impression was that providers are not happy to provide care to women. There is not really good contact between women and providers. More than 80% of our informants had no education; so, it is impossible for them to read what is mentioned on the visit card. When they do a delay about an appointment, the care providers shouted them in the presence of the other women. Some users were very humiliated. The appointment for immunization is often difficult to be remembered by women therefore the techniques of calculation with reference to the days of market or Friday for Muslim and Sunday for Christian. In one of the districts, the auxiliary midwife did not speak the local language; so it was very difficult for her to discuss with or to be understood by women. “*There are some kind midwives and also some, who are not kind. If you meet them, they will shouted at you « can't you do this, can't you put up with pain, you move a lot, you did not know, when you made love with your husband? Was I there?* ”. FGD, Village C.

It was reported in FGDs that some women delivered on the floor in the labour room. This happened because the auxiliary midwife was in her house instead of being in the health facility. According to some informants, when they call the midwife, some times, she shouted at them and tells them that the labour has a long way to go and then the baby appears and the woman delivered on the floor. The woman delivers alone and after the auxiliary midwife comes for the others care and then she shouted at her.

### Distance

In both district, distance and accessibility are evoked as barriers to use of maternity services. The terrain is uneven and the roads condition is bad. To reach the maternity, there is often no means of transport and when there is one, the cost is high. Usually, women go to maternity by foot or by bicycle or motorcycle. When the labour begins at home and the woman has to walk five or more than five kilometres, it is not easy. Also during, the rainy season, the roads are impassable. Sometimes, there is a river to cross before reaching the maternity.

The problem of distance was more mentioned in the district of Ouargaye. Accessibility in this district is more difficult. Some women stay at home because they are too far from a health centre. Those who have a bicycle or the husband has a motorcycle can be taken to the maternity if the others conditions are okay. “*Those who delivered at home, some time don't go to maternity after. It is especially those who are in remote villages, very distant from the health centre. If they don't have bicycle to go to maternity, how will they do? Also, it is difficult to walk when you are pregnant; in this situation, you are obliged to stay at home until delivery*” FGD, Village A.

## Discussion

The perception of bad care is related to misunderstanding of some care. In most of the time, providers didn't inform users about their illness and did not take time to discuss with them. A few questions are asked to them and then a prescription is written. This situation seems to be general; there is lack of communication with users in health facilities [12]

Reception contributed to good perception of quality of care; but reception depends on many factors. In Ouargaye district, the SCI had around 12 training sessions to improve skills of providers involved in obstetric care. Some had training in COPE (Client-Oriented, Providers-efficient) to improve quality of relationship with clients. Normally with this training, the client is put forward and attention is paid to him. In reality, reception depends on the professional integrity and the relationships between providers and the community.

Another aspect of perception of quality of care is referral. In both districts, referral is perceived as the same. It happened when it is not possible for staff to do more in the local health facility. In this case, the problem is to find a transport mean to reach the district or regional hospital. In the national health policy, it is not possible to do surgery in the local health facility; this situation is not well perceived by women who considered it as a lack of equipment and competences for staff. Referral increases charges for the family. For referral, it is possible to use the ambulance but it is not always available. There is only one ambulance for the whole district.

When death occurs in maternity before or after referral, it is perceived differently. For relatives, it is a kind of destiny because they have done their best by seeking care.

Intervening conditions preventing for use of skilled care in both districts are constraining for women. But the cultural practices evoked in Ouargaye are not really a problem for use of maternity; it concerns the young women with first-time pregnancy and leads some times to delay in the use of health facility. Before getting permissions, [13] pregnancy must be announced to the community around the third or fourth or fifth month of the pregnancy. In Diapaga, cultural beliefs don't always allow the use of health facility. It is a kind of opposition between tradition and modernity. The local knowledge is not to be abandoned but soothsayers and traditional healers could be sensitized on health problems and specifically on pregnancy and its complications.

The difficulty to reach a health facility is still a problem in the two districts. According to the ministry of health, the mean distance to a health facility must be 9 kilometres. In Ouargaye, this mean distance is 8, 85 and 14, 97 for Diapaga [2]. But in reality, these distances seem long due to the terrain which is uneven and hilly in both districts. In the rainy season, the use of maternity services decreases. People used to stay in the fields during this period. The distance to reach a health centre is multiplied some time by two or three. In these conditions, they cannot follow antenatal care and deliver in most of time at home.

Financial problem was not specific to a district; in Ouargaye, the SCI had contributed to make free some services in maternity. Charges for visit card and some medicines (iron) were waived while in Diapaga these things have to be bought by women. There is in both districts the cost-sharing system but this system did not concern all the care. It concerned only cost for surgery when there is complication. This system could be generalized for all care. Waiver of charges for care could be also a solution to increase the use of health facilities.

Many studies in different developing countries have evoked bad treatments that women can receive in health facilities [14]. These treatments lead to the choice to stay at home with the traditional birth attendance or other unskilled persons to deliver with potential risk of complication.

## Conclusion

Nowadays, the general tendency is to use maternity services to benefit from skilled care. This situation is favoured by sensitizing that providers do during care and also when they visit the communities for immunization and for discussion on health. This is a general aspect but the use of skilled care depends on the national policy and the role of the districts managers. There is not a common policy to increase the use of maternity services. In the study districts, the perception of quality of care is positive even there are many things to do to increase the supply of care and the choice for women who needed skilled care. This good appreciation can be perceived as a bias due to the fact that researchers in the domain of health are generally considered as health provider. The advantages described by women of using maternity services could be used by district managers and providers for sensitizing. These elements are the more important reasons for women to use maternity services. The barriers described are common to many districts [6]. Those related to cultural factors could be more difficult to change but those concerning the health system could be avoided by policy makers by improving the supply of care and motivating providers more and could be amenable to education in being sensitive.
